# A Study of Occurrence of Musculoskeletal Discomfort in Computer Operators

**DOI:** 10.4103/0970-0218.39252

**Published:** 2008-01

**Authors:** Dinesh Bhanderi, SK Choudhary, Lata Parmar, Vikas Doshi

**Affiliations:** Department of Community Medicine, Pramukh Swami Medical College, Karamsad, Anand, Gujarat, India

## Introduction

Technological advances, particularly, invention of computers, have revolutionized our way of working. Computer has become an integral part of our life. However, its use is not free from health hazards. Intensive computer work puts stress and strain on muscles, as well as joints, because of continuous and repetitive nature of movements. Individual factors, prolonged awkward postures, poor workstation design and psycho-social environment can lead to development of symptoms of musculoskeletal discomfort (MSD). If these symptoms are ignored and if no preventive measures are taken, cumulative trauma disorders such as myalgia, myofascial syndromes, nerve entrapment syndromes, tendonitis, epicondilitis and tenosynovitis can develop.(1) In this paper, which is a part of a broader cross-sectional study, 419 individuals using computers were studied, and a number of individual factors of presumed importance were investigated.

## Materials and Methods

The study was conducted in two *talukas* of Anand district - namely, Anand and Petlad - from May 2004 to January 2006. The sample size of 44 was calculated considering a prevalence of computer-related health problems as 20%, and 10% non-response rate. Thus 440 was the final sample size. Establishments and institutes in Anand and Petlad *talukas,* where computers are extensively used, were enlisted. These included banks, computer training centers and colleges running degree courses in computer applications. After seeking the permission of the heads of these institutes, all the staff and faculty members, as well as final year students, were enlisted. Four hundred forty subjects were selected randomly. Thirty-one did not participate in the study, making the nonresponse rate of 7.0%. Rest of the subjects (n = 419) were asked to fill up a pre-tested questionnaire, after obtaining their verbal consent. Other relevant information was gathered through personal inspection of workstation. Musculoskeletal discomfort (MSD) was considered when one or more of the following symptoms were reported by the respondents: neck or shoulder stiffness; neck or shoulder pain; tingling/numbness in hands, thumbs or fingers during work or many hours after stopping work; hand and wrist pain; backache; headache; leg cramps; leg stiffness; numbness in ankles and feet; swelling in ankles and feet; reduction in strength of hand and difficulty in grasping objects.

## Results and Discussion

Mean age of the subjects who participated in this study was 25 years, with a range of 18-55 years. Three-fourths of the subjects were young, with age of 15-25 years; and 279 (66.6%) were male. Majority (65.4%) of the respondents started using computers at a young age (16-20 years), and 236 (56.3%) individuals had been using computers for less than 5 years. About 41% of the respondents used to work on computers for about 21 to 40 hours in a week.

The prevalence of self-reported symptoms related to MSD ranged from 0.7-34.8%. Overall prevalence of any MSD was 75.2% (315/ 419). Armstrong *et al.* reported MSD prevalence of 25-76% among video display unit (VDU) operators in his study.(2)

Individual factors like age and gender play a significant role in causing MSD.(3) Similar findings were reported by Demure *et al.*(4) and Karlquist *et al.*(5) in their studies. However, age and gender were not linked with any of the MSD studied by Ortiz *et al.*(6) Our study did not find any association between age of the subject and occurrence of MSD [Table 1]. Slightly higher proportion of female subjects reported MSD. However, it was not found to be statistically significant.

**Table 1:** Distribution of occurrence of musculoskeletal discomfort with background variables

Variable	Musculoskeletal discomfort		
		
	No No. (%)	Yes No. (%)	Total No. (%)
Age of the subject (years) χ^2^ = 3.158, df = 3, *P* > 0.05			
15-25	84 (26.1)	238 (73.9)	322 (100.0)
26-35	15 (23.4)	49 (76.6)	64 (100.0)
36-45 5	(20.8)	19 (79.2)	24 (100.0)
46-55	0 (0.0)	9 (100.0)	9 (100.0)
Gender χ^2^ = 0.176, df = 1, *P* > 0.05			
Female	33 (23.6)	107 (76.4)	140 (100.0)
Male	71 (25.4)	208 (74.6)	279 (100.0)
Habit of doing exercise χ^2^ = 0.007, df = 1, *P* > 0.05			
No	52 (25.0)	156 (75.0)	208 (100.0)
Yes	52 (24.6)	159 (75.4)	211 (100.0)

Two hundred eleven (50.4%) subjects enrolled in our study were doing one or more physical exercises like walking, running, etc. Such physical activity was not found to be associated with MSD in our study [Table 1]. A study by Korhonen *et al.*(7) showed that computer users doing no or less physical exercise are at higher risk of MSD related to neck.

Habit of taking breaks for rest has been found to be associated with occurrence of MSD.(3,4,7) However, some studies(8) contradict this by showing no relationship between rest breaks and occurrence of MSD. Our study also did not observe any association between habit of taking breaks and occurrence of MSD [Table 2].

**Table 2:** Distribution of occurrence of musculoskeletal discomfort with other variables

Variable	Musculoskeletal discomfort		
		
	No No. (%)	Yes No. (%)	Total No. (%)
Habit of taking breaks χ^2^ = 0.540, df =1, *P* > 0.05			
No	34 (27.2)	91 (72.8)	125 (100.0)
Yes	70 (23.8)	224 (76.2)	294 (100.0)
Duration of computer use (hrs/week) χ^2^ = 24.286, df =3, *P* < 0.001			
<20	49 (40.5)	72 (59.5)	121 (100.0)
21-40	27 (15.7)	145 (84.3)	172 (100.0)
41-60	22 (21.4)	81 (78.6)	103 (100.0)
>60	6 (26.1)	17 (73.9)	23 (100.0)
Refractive error χ^2^ = 6.094, df = 1, *P* < 0.05			
No	77 (28.7)	91 (71.3)	268 (100.0)
Yes	27 (17.9)	124 (82.1)	151 (100.0)

A dose response relationship has been reported between duration of computer use and prevalence of musculoskeletal problems in literature.(3,4,5,9) Our study showed highly significant association between MSD and duration of computer use [Table 2]. However, Ortiz *et al.* did not observe such association in their study.(6)

In our study, vision of 269 (64.2%) subjects was normal, while 141 (33.7%) suffered from myopia, 4 (1.0%) from presbyopia and 5 (1.2%) from both. A significantly higher proportion of respondents with refractive error reported occurrence of MSD [Table 2]. Studies have demonstrated that sustained work with VDUs leads to temporary myopization of vision.(10) Therefore, the subject may deviate from the neutral posture to get a clear vision of the screen. This deviation may cause MSDs.

Thus, our study suggests that MSDs are a common problem among those who use computer intensively. It seems likely that long hours of working on computers and presence of refractive error may result in these problems. Future longitudinal studies should evaluate the relative importance of other individual factors like age, gender, habit of doing exercise and taking breaks. Results from such epidemiological studies can contribute to the development of appropriate hazard-prevention programs for workers who frequently use computers.
